# Draft genome of a manganese-oxidizing bacterium *Marinobacter* sp. DUT-1 with potential metal resistance

**DOI:** 10.1128/mra.00510-25

**Published:** 2025-10-09

**Authors:** Jieyi Li, Tongtong Wu, Hao Zhou, Hongzhi Tang, Haixia Pan

**Affiliations:** 1 Key Laboratory of Industrial Ecology and Environmental Engineering (Ministry of Education), School of Chemical Engineering, Ocean and Life Sciences, Panjin Campus, Dalian University of Technology, Panjin, China; 2 Guangxi Key Laboratory of Beibu Gulf Marine Resources, Environment and Sustainable Development, Fourth Institute of Oceanography, Ministry of Natural Resources, Beihai, China; University of Southern California, Los Angeles, California, USA

**Keywords:** manganese-oxidizing bacteria, *Marinobacter*, genome sequencing, marine sediments

## Abstract

The marine bacterium *Marinobacter* sp. DUT-1, isolated from Bohai Bay surface sediment, exhibits Mn(II) oxidation capability. Its genome contains 4,004,237 bp of sequence with a GC% content of 59.17. The isolation of this manganese-oxidizing bacteria highlights its potential role in metal pollution remediation in marine environments.

## ANNOUNCEMENT

Severe contamination of heavy metals, such as Pb, Cd, Zn, and Mn, has been found in Bohai Bay (China) coastal areas (1, 2). Manganese-oxidizing bacteria can oxidize Mn(II) to biogenic manganese oxides (BioMnOx). BioMnOx with high oxidative and adsorption capacity has been applied in heavy metals removal such as Cr, Co, and Se (3).

We isolated a Mn(II)-oxidizing bacterium, DUT-1, from nearshore surface sediments of Bohai Bay (121.55°E, 40.75°N). Purification of strain DUT-1 used a modified neutral haloarchaeal medium with the following composition (per L): yeast extract 0.05 g, peptone 0.25 g, sodium pyruvate 1.0 g, KCl 5.4 g, NaNO_3_ 0.429 g, MgSO_4_·7H_2_O 13.4 g, MgCl_2_·6H_2_O 11.5 g, NaCl 35.0 g, K_2_HPO_4_ 0.3 g, and CaCl_2_ 0.29 g (pH 7.0–7.2) (4). To prevent precipitation, separate filter-sterilized (0.22 µm) solutions of K_2_HPO_4_, CaCl_2_, and MnCl_2_·4H_2_O were aseptically added. Primary isolation employed spreading of 100 µL sediment suspension (1 g in 9 mL saline) onto solid medium (20 g/L agar) of this composition. During 7 days of incubation at 30°C, the colonies that turned blue when reacting with the leucoberbelin blue solution were selected and incubated in fresh liquid medium (5). Pure isolates were obtained through at least three cycles of alternating subculturing, comprising transfer from liquid medium to solid medium for streak-plate purification, followed by a return to liquid culture.

The strain DUT-1 was incubated in modified medium with 1.0 g/L yeast extract at 30°C for 4 days before PCR amplification of the 16S rRNA gene and draft genome sequencing (6). Genomic DNA was extracted using a modified cetyltrimethylammonium bromide (CTAB) method. Using the Rapid Plus DNA Lib Prep Kit for Illumina (RK20208), the DNA underwent end repair, A-tailing, adapter ligation, size selection, and PCR amplification prior to sequencing on the Illumina NovaSeq 6000 platform (PE150). Fastp (v1.0.1) filtering of 13,457,788 raw reads (2,018.66 Mb) yielded 13,401,530 clean reads (2,010.22 Mb). The clean data were individually assembled by SOAPdenovo (v2.04; k-mers 95,107,119), SPAdes (v4.1.0; k-mers 99,127), and Abyss (v2.3.10; k-mer 64), followed by integration using CISA software with stringent parameters (95% identity, 50 bp overlap), with conflicts resolved by >10× coverage priority and subsequent removal of contaminants (<0.35×) and fragments <500 bp (7–9). Default parameters were used for all software unless otherwise specified in this study. To annotate manganese-oxidizing genes and metal resistance genes (MRGs), a comprehensive tool (MnOxGeneTool) and MRG database were used (10, 11). The identification of strain DUT-1 based on 16S rRNA and genome sequence was performed at EzBioCloud and Type Strain Genome Server (TYGS) (12, 13).

According to EzBioCloud analysis of the 16S rRNA gene sequence obtained by PCR amplification, strain DUT-1 shows a 99.3% similarity with *Marinobacter pelagius* HS225 (GenBank accession no. NR_043863). Based on TYGS analysis, the dDDH (d4) value was 41.7% between strain DUT-1 and *Marinobacter pelagius* CGMCC 1.6775 (GenBank accession no. GCA_900114925). Therefore, strain DUT-1 was a novel species of genus *Marinobacter*. Genome analysis showed that the genome contains 14 contigs with a total length of 4,004,237 bp (N50 = 963,140 bp), GC content of 59.17% and sequencing coverage of 528×. Genome annotation was using Prokaryotic Genome Annotation Pipeline with 694 protein-coding genes and 48 tRNAs, 9 rRNAs (including 4 × 5S, 2 × 16S, and 3 × 23S) were obtained. Functional annotation revealed two genes encoding a multicopper oxidase (*copA*) and a KatG-family catalase (*stKatG*), respectively, which are probably involved in manganese oxidation. Meanwhile, several MRGs were found in the genome of strain DUT-1, for instance, *copA*, *merA*, *cusA/ybdE,* and *czcA*. The draft genome of *Marinobacter* sp. DUT-1 provides insight into manganese-oxidizing and metal-resistant mechanisms in marine bacteria.

## Data Availability

The 16S rRNA sequence of *Marinobacter* sp. DUT-1 was submitted to GenBank database under accession number PV384101. The draft genome sequence was deposited in SRA and GenBank under accession number SRR32868335 and JBNRLS000000000, respectively (BioProject PRJNA1242079 and BioSample SAMN47579242).
